# Novel Superelastic Polyesters Based on 2,5-Furandicarboxylic Acid for Potential Use in Ophthalmic Surgery

**DOI:** 10.3390/polym17233220

**Published:** 2025-12-03

**Authors:** Arianna Palumbo, Gloria Astolfi, Giulia Guidotti, Michelina Soccio, Elisa Boanini, Piera Versura, Nadia Lotti

**Affiliations:** 1 Department of Civil, Chemical, Environmental and Materials Engineering (DICAM), University of Bologna, Via Terracini 28, 40131 Bologna, Italy; arianna.palumbo3@unibo.it (A.P.); m.soccio@unibo.it (M.S.); nadia.lotti@unibo.it (N.L.); 2 Ophthalmology Unit, Dipartimento di Scienze Mediche e Chirurgiche (DIMEC), Alma Mater Studiorum Università di Bologna, 40126 Bologna, Italy; gloria.astolfi2@unibo.it (G.A.); piera.versura@unibo.it (P.V.); 3 Department of Chemistry “Giacomo Ciamician”, University of Bologna, Via Gobetti 85, 40129 Bologna, Italy; elisa.boanini@unibo.it

**Keywords:** poly(pentamethylene furanoate), poly(hexamethylene furanoate), physical blends, copolymerization, superelasticity, mechanical recovery, corneal surgery, cell viability

## Abstract

The rapid development of ophthalmic surgery in recent years has made big steps forward, making interventions such as penetrating and lamellar keratoplasty or trabeculectomy widely practiced. However, the use of non-absorbable sutures in these procedures poses significant challenges. Indeed, unequal tension between the various stitches can lead to deformations of the cornea or lens and consequently to problems such as post-operative astigmatism or anisometropia. To overcome these problems, sutures with improved closure via a highly stretchable behaviour together with an excellent elastic return are a credible solution. Accordingly, to widen the plethora of superelastic polymeric materials, in the present study a novel solution deriving from two furan-based polyesters, poly(pentamethylene furanoate), PPeF, and poly(hexamethylene furanoate), PHF, was successfully obtained. Of note, these homopolymers are also entirely derived from sustainable sources. The two homopolymers were physically and chemically mixed to obtain copolymers with different block lengths, which were characterised from molecular, thermal, mechanical, and surface wettability points of view, showing interesting properties which were easily modulated as a function of block length. Lastly, all the materials showed good stability over time and cell viability and, for some of them, a great mechanical recovery upon deformation was also observed.

## 1. Introduction

Shape memory alloys (SMAs), particularly those based on near-equiatomic nickel–titanium compositions known as Nitinol, have had a significant impact on biomedical engineering [1]. Their unique combination of superelasticity and shape memory effects [2], enabled by reversible, diffusionless, solid–solid phase transformations between the high-symmetry austenitic phase (B2) and the low-symmetry martensitic phase (B19′ monoclinic), has allowed these materials to recover from strains of up to 8%. This renders them suitable for a broad array of dynamic biomedical applications [3,4,5,6,7,8]. Nitinol has thus become the material of choice for several implantable devices, including endovascular stents, orthodontic archwires, vena cava filters, and microsurgical staplers used in minimally invasive procedures [4]. In ophthalmology, it has been incorporated into devices for precise deployment and stabilisation, benefiting from its elasticity and thermal responsiveness [9].

Despite its considerable advantages, the use of Nitinol is not without limitations. These include the potential for nickel ion release and allergic responses, as well as progressive loss of pseudoelasticity due to accumulated residual strain and dislocations at the martensite–austenite interfaces during cyclic loading [10,11,12,13,14]. While Nitinol is generally biologically compatible and inert from a cytotoxic point of view, its metallic nature can present challenges in certain surgical contexts, such as ophthalmic procedures, where transparency, low weight, and controlled biodegradation are highly desirable [9,13,15]. These challenges have sparked growing interest in next-generation materials that combine no-cytotoxicity with mechanical adaptability and functional intelligence.

One promising area of research is the use of smart polymeric materials, especially bio-based or bioengineered polymers, for microsurgical tools and implants [16,17]. In ophthalmology, smart polymeric systems have already been successfully used in devices such as glucose-responsive contact lenses [18,19], offering a glimpse into a future where surgical implants serve not only structural functions, but also assist in diagnostics and therapy.

Moreover, biopolymers offer several advantages over metallic shape memory alloys (SMAs). They typically exhibit lower stiffness, which reduces the risk of stress shielding—a phenomenon associated with metallic implants such as those made of titanium [20]—and they are less likely to provoke immune or inflammatory responses [21]. Their intrinsic flexibility and transparency also make them appealing for use in microscale ophthalmic interventions, such as intraocular or periocular implants, where precision, minimal invasiveness, and tissue compatibility are paramount [22].

Accordingly, in the present study, novel materials derived from 2,5-furandicarboxylic acid (FDCA) have been investigated for the first time for potential applications in ophthalmic microsurgery. FDCA is a bio-based analogue of terephthalic acid [23], the monomeric precursor of polyethylene terephthalate (PET), a polymer which is already widely used in biomedicine [24,25], i.e., sutures [26], artificial ligament grafts [27], ureteral stents [25], and graft vein in vascular bypass surgery [25]. FDCA is also a sustainable building block for the synthesis of aromatic polyesters, which is in line with the increasing demand for environmentally friendly materials also in the biomedical field.

Among the furan-based polymers already investigated in the literature, very few examples are reported about biomedical applications [23,28,29,30,31], and none of them are about materials which combine high flexibility and elasticity [32,33] with good cell viability. Accordingly, in the present study, particular attention was given to poly(pentamethylene 2,5-furanoate) (PPeF), which exhibits a soft, elastomeric mechanical response thanks to its flexible aliphatic glycolic subunit and lack of crystallinity [34,35,36]. However, its low tensile strength and limited mechanical resistance are challenging for applications involving mechanical stresses such as those encountered in microsurgical ophthalmic environments.

To address these limitations, PPeF has been physically blended, in the same weight amount, with poly(hexamethylene 2,5-furanoate) (PHF), a semicrystalline and mechanically rigid homopolymer [34,35]. PHF was specifically chosen also because of its chemical structure, similar to the one of PPeF, which promotes thermodynamic compatibility and potential miscibility with PPeF—a necessary requirement for achieving homogeneous phase behaviour and stable material properties [37]. The resulting blends are expected to show both the elastomeric flexibility of PPeF and the mechanical strength of PHF, together with tunable shape recovery, suitable for smart biomedical systems.

Copolymers of PPeF and PHF with varying block lengths were also synthesised, to investigate the impact of molecular architecture on physico-chemical and functional behaviour of the resulting copolyesters. In particular, all synthesised materials were comprehensively characterised from molecular, structural, morphological, and thermal points of view. Particular emphasis was placed on mechanical testing, to evaluate the elastic modulus, tensile behaviour, and shape recovery potential of the materials. Last, in vitro cytotoxicity tests were conducted to assess cell viability. Although some blends and/or copolymers of PPeF [38,39,40,41] and PHF [42,43,44,45] have been already investigated in the literature, to the best of our knowledge, it is the first time that these two homopolymers have been properly combined together to obtain materials with tuned and suitable elasticity and able to support ophthalmic cell growth. The results obtained also suggest that these FDCA-based polymers have the potential to be used as next-generation smart materials in minimally invasive and not cytotoxic ophthalmic devices, as sustainable and functional alternatives to metallic SMAs, thus widening the list of possible applications of furan-based polyesters.

## 2. Materials and Methods

### 2.1. Materials

Dimethyl furan-2,5-dicarboxylate (DMF) was purchased from Sarchem Labs (Farmingdale, NJ, USA purity of 99.8%). 1,5-Pentanediol (PD) (purity of 97%), titanium tetrabutoxide (TBT), titanium isopropoxide (TIP), and chloroform (purity ≥ 99.8%) were purchased from Sigma-Aldrich (Saint Louis, MO, USA). The sample of 1,6-Hexanediol (HD) (purity ≥ 97.0%) was purchased from TCI (Zwijndrecht, Belgium). All reagents were used without further purification.

### 2.2. Synthesis of Homopolymers

**Scheme 1:**
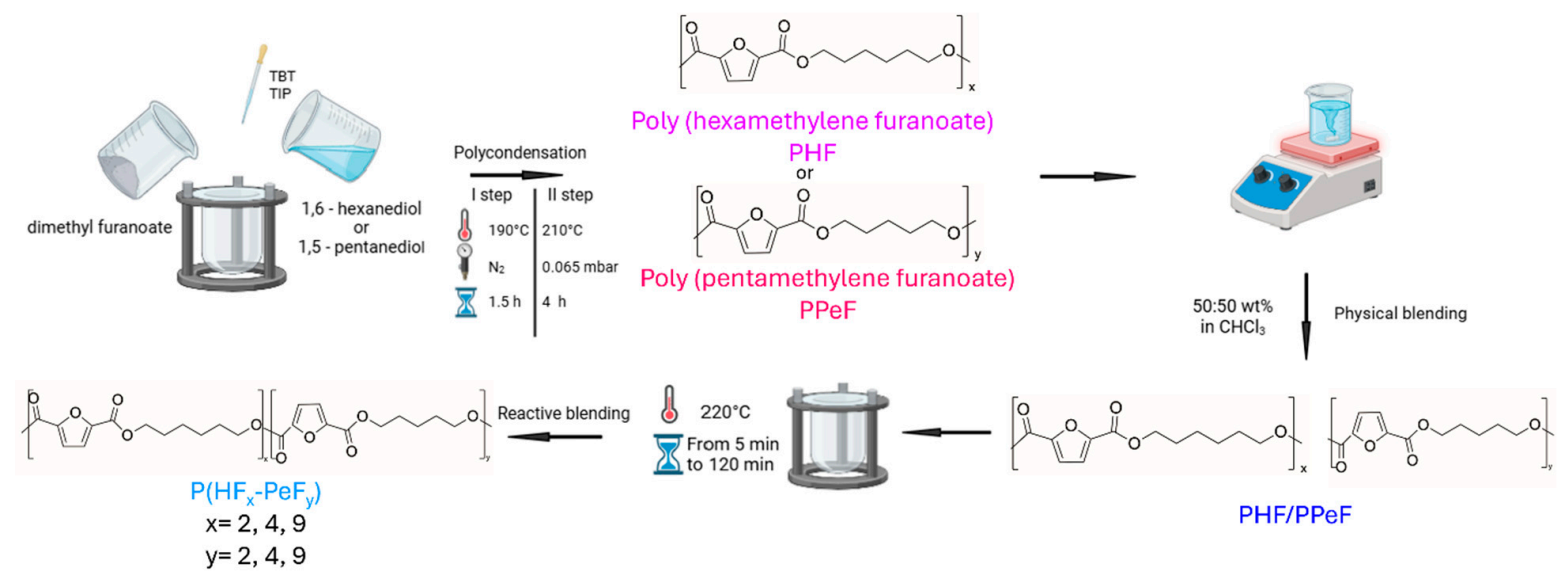
Schematic representation of the synthetic conditions leading to the production of PPeF and PHF homopolymers, the physical blend, and the copolymers under study, together with their chemical structures.

Poly(pentamethylene 2,5-furanoate) (PPeF) and poly(hexamethylene 2,5-furanoate) (PHF) were synthesised using a two-step bulk polycondensation process (Scheme 1). For PPeF, the reagents dimethyl furan-2,5-dicarboxylate and 1,5 pentanediol were charged in a thermostatted stirred glass reactor with a 1:2 molar ratio, while for PHF, DMF and 1,6 hexanediol were charged using the same glycolic excess. The catalysts TBT and TIP were added at a concentration of 200 ppm each. The first step was carried out in an inert N_2_ atmosphere with continuous stirring (50 rpm), at 190 °C and under room pressure. During this stage, transesterification reactions took place releasing methanol, which was removed from the reaction environment by distillation. The stage was considered complete once 90% of the theoretical amount of methanol was collected (about 90 min). In the second step, the reaction system was gradually heated to 210 °C and the pressure was simultaneously reduced to 0.065 mbar. When high and constant torque value was measured (about after 4 additional hours), the polymer was discharged from the reactor and stored at room temperature.

### 2.3. Blend Preparation

Following the synthesis, a binary blend, namely PHF/PPeF, was prepared starting from the same weight amount of the two homopolymers. PPeF and PHF were dissolved under stirring at room temperature in the minimum amount of chloroform plus some drops of hexafluoroisopropanol. The resulting solution was then transferred to a Petri dish to allow the solvent to evaporate.

### 2.4. Copolymer Synthesis

Block copolymers were synthesised from the PHF/PPeF physical mixture through reactive blending in a glass reactor under nitrogen atmosphere at 220 °C. To obtain copolymers with different block lengths, different mixing times (5, 10, and 120 min) were selected. The samples were indicated as P(HF_x_–PeF_y_), where x and y represent the block lengths, as determined by ^13^C-NMR.

### 2.5. Film Preparation

Thin films with a thickness of about 100 micrometres were prepared by compression moulding using a Carver C12 press (Wabash, IN, USA). The homopolymers, the physical blend, and the copolymers were placed between two Teflon sheets and heated to 20 °C above the respective melting temperature (or glass transition temperature in the case of amorphous PPeF). Once molten, a pressure of 6 tons/m^2^ was applied for 2 min. The films were then cooled to room temperature and stored for three weeks to attain equilibrium crystallinity, before further characterisation.

### 2.6. Molecular Characterisation

The chemical structure and composition were determined by ^1^H-NMR spectroscopy (Varian INOVA 400 MHz, Palo Alto, CA, USA), while the degree of randomness (R) and block length (L) were determined by ^13^C-NMR spectroscopy (Bruker 600 MHz, Billerica, MA, USA). The samples were dissolved in deuterated chloroform with tetramethylsilane (TMS, 0.03 vol%) as internal standard at a concentration of 10 mg/mL for ^1^H-NMR and 30 mg/mL for ^13^C-NMR, respectively. Measurements were carried out at 25 °C.

The molecular weights (M_n_) and corresponding polydispersity indexes (Ð) were evaluated at 25 °C using gel permeation chromatography (GPC) with an HPLC Lab Flow 2000 apparatus (Waters, Milford, MA, USA) equipped with a Rheodyne 7725i injector (Thermo Fisher Scientific, Waltham, MA, USA), a Phenomenex Phenogel MXM 5 µm mixed-bed column (Torrence, CA, USA), and an RI K-2301 detector (KNAUER, Berlin, Germany). The instrument was calibrated using polystyrene standards with molecular weights ranging from 550 to 2,500,000 g/mol. The eluent was HPLC-grade chloroform, with a flowing at a rate of 1 mL/min. The samples were prepared by dissolving the homopolymers in the same solvent (2 mg/mL).

### 2.7. Thermal Analysis

The thermal stability of the prepared samples was investigated by means of thermogravimetric analysis (TGA) with a PerkinElmer TGA4000 instrument (Waltham, MA, USA). Measurements were taken under an N_2_ flow of 40 mL/min, with the polymer (8–10 mg) being heated from 40 to 800 °C at a rate of 10 °C/min. The initial degradation temperature (T_onset_) and the temperature corresponding to the maximum degradation rate (T_max_) were determined.

Differential scanning calorimetry (DSC) was used to identify the main thermal transitions of polymer samples subjected to a specific thermal protocol. The experiments were performed using a Pyris DSC6 calorimeter (Waltham, MA, USA) under a nitrogen atmosphere at a flow rate of 20 mL/min. First, approximately 5–8 mg of polymer film was heated from −40 °C to 180 °C at a rate of 20 °C/min (first heating scan), held isothermally for one minute, and then cooled rapidly back to −40 °C at 100 °C/min. A second heating cycle was then carried out from −40 °C to 180 °C, again at a rate of 20 °C/min (second heating scan). The glass transition temperature (T_g_) was determined as the midpoint of the transition from the glassy to the rubbery state. The change in specific heat capacity (ΔC_p_) was calculated based on the step height between the two baselines that define the glass transition region. The melting temperature (T_m_) was identified at the peak of the endothermic melting transition, while the melting enthalpy (ΔH_m_) was determined from the total area under the relative endotherm. Similarly, the cold crystallisation temperature (T_cc_) and the associated enthalpy change (ΔH_cc_) were evaluated from the DSC thermograms.

### 2.8. Structural and Morphological Characterisation

The nature and quantity of crystalline phases were determined by means of wide-angle X-ray scattering (WAXS), employing a PAN analytical X’PertPRO diffractometer (Almelo, The Netherlands) equipped with an X’Celerator detector and a copper target. Analysis was conducted at room temperature within the 3–60° 2θ range (acquisition time: 100 s/step; step size: 0.10°). The degree of crystallinity of each sample was determined by calculating the ratio of the area under the crystalline peak to the total area under the diffractometric curve.

The investigation of the microstructure and morphology of the sample was conducted using a Zeiss Leo-1530 (Oberkochen, Germany) scanning electron microscope, with an accelerating voltage of 5 kV (secondary electrons). The cryo-fractured cross-sections of the polymeric films were analysed post-gold metallisation through physical vapour deposition (PVD).

### 2.9. Water Contact Angle Measurements

Static water contact angle (WCA) measurements were taken on flat film surfaces using a Krüss DSA30S instrument (Hamburg, Germany) with Drop Shape Analysis software (Version 1.92.1.1). Prior to testing, each sample was cleaned with a 70% (*v*/*v*) ethanol/water solution and left to dry overnight at room temperature. Droplets of deionised water (volume of 4 µL each) were dispensed onto each film and their profile recorded one second after placement. The contact angle was then determined using the analysis software. Ten measurements were taken for each sample, and the final WCA value was expressed as the mean ± standard deviation.

### 2.10. Mechanical Characterisation

The mechanical properties of polymeric films (5 × 50 mm^2^, gauge length of 20 mm) were determined using an Instron 5966 dynamometer (Norwood, MA, USA) equipped with a rubber grip and a transducer-coupled 10 kN loading cell which is controlled by a computer. The tests were performed at room temperature and 50% relative humidity with a strain rate of 10 mm/min until the specimen breaks. The tensile elastic modulus (E) was determined from the initial linear slope of the obtained stress–strain curves. Elongation at break (ε_b_) and stress at break (σ_b_) were also determined from these curves. The results obtained are reported as the average value ± standard deviation of at least five measurements for each sample. The elastic return was evaluated using a cyclic loading analysis. To achieve 6% and 20% elongations, samples without yielding points were strained and released for 20 cycles, at a rate of 10 mm/min.

### 2.11. Hydrolytic Degradation Tests

To confirm the stability of the materials under physiological conditions, hydrolytic degradation tests were carried out on PHF/PPeF blend and P(HF_2_–PeF_2_) copolymer.

The experiments were performed by incubating polymeric films (5 × 60 mm), previously weighed, in phosphate-buffered saline solution (pH = 7.4) at 37 °C inside a shaking incubator (Stuart SI500, Cole-Parmer Ltd., Staffordshire, UK). The buffer solution was periodically changed to keep the pH constant. After six months, triplicate specimens for each sample were recovered from the incubator, repeatedly washed with deionised water, and dried for 2 days to reach constant weight. Weight loss was determined by comparing the residual dry weight at a specific time with the initial weight. Molecular weight loss data were obtained by gel permeation chromatography (GPC) at 30 °C; thermal and mechanical properties of partially degraded samples were studied as previously described.

### 2.12. In Vitro Citotoxicity Evaluation

Prior to each test, the homopolymers PHF and PPeF, the physical blend PHF/PPeF, and the random copolymer P(HF_2_–PeF_2_) were purified by dissolving them in the minimum amount of chloroform, before reprecipitating them in a large excess of methanol. Small, square sections of the films obtained from purified samples (approximately 0.5 cm^2^ each) were subjected to a two-step ethanol treatment to clean the surface. First, the samples were placed in a 90% ethanol solution for 30 min and then transferred to a 70% ethanol solution for further 30 min. Last, films were then thoroughly washed with phosphate-buffered saline at pH 7.4 to remove any residual ethanol.

Human corneal fibroblasts (HCornF, P10872; Innoprot (Zamudio, Biscay, Spain)), were seeded in 24-well plates (with or without films) at a density of 2 × 10^4^ cells per well, and in 96-well plates at a density of 5 × 10^2^ cells per well. The cells were cultured in DMEM (Gibco, Fisher Scientific, Hampton, NH, USA), supplemented with 10% FBS, 1 mM L-glutamine, and antibiotics (penicillin at 20,000 U/mL and streptomycin at 20,000 µg/mL, both from Lonza Group Ltd., Basel, Switzerland), at 37 °C in a 5% CO_2_ incubator. Passages 1–5 were used. Cell growth, morphology, and confluency were monitored daily using an inverted microscope (Zeiss Axiocam 208, Oberkochen, Germany).

Cytocompatibility was assessed in accordance with ISO 10993-5 guidelines [46], employing both direct contact and extract-based methods. For the 3-(4,5-dimethyl-2-thiazolyl)-2, 5-diphenyl-tetrazoluim bromide (MTT) assay, cell viability was evaluated after 24, 48, and 72 h by incubating the samples in a 0.5 mg/mL MTT solution at 37 °C for two hours, followed by solubilising the formazan with isopropanol. Absorbance was then measured at 570 nm using a Multiskan SkyHigh plate reader (Thermo Fisher Scientific, Waltham, MA, USA). For the direct contact test, 0.5 cm^2^ material fragments were placed on subconfluent monolayers of HCornF cells in 24-well plates. Incubation was carried out at 37 °C with 5% CO_2_ for up to 72 h. For the extract test, polymer samples, including commercial PBS, PHF, and PPeF homopolymers, PHF/PPeF blend, and P(HF_2_–PeF_2_) copolymer were incubated in DMEM containing 5% FBS and 1% antibiotics for 24 h. Then, extracts (100 µL) were applied to HCornF cells cultured in 96-well plates under the same conditions as the direct contact assay. All data represent the mean ± standard deviation from three independent experiments performed in triplicate. One-way ANOVA (GraphPad Prism 10.0) was used for statistical analysis, with significance set at *p* < 0.05.

## 3. Results and Discussion

### 3.1. Molecular Characterisation

Magnetic resonance spectroscopy was used to confirm the chemical structure of all the polymers presented in this study, excluding the presence of secondary reactions that could significantly alter their chemical composition and/or molecular architecture. These structures were validated by observing proton (^1^H-NMR) and carbon (^13^C-NMR) spectra.

**Figure 1:**
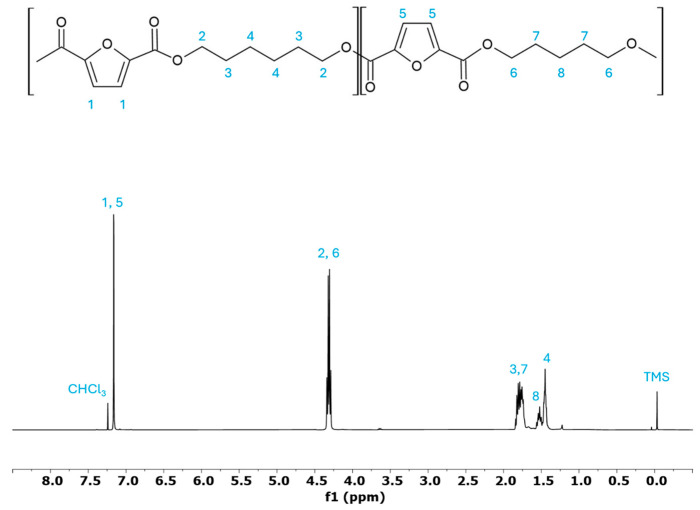
^1^H-NMR spectrum of the copolymer P(HF_9_–PeF_9_) with signal assignment.

As to ^1^H-NMR, in Figure 1, the spectrum of the P(HF_9_–PeF_9_) copolymer is shown, together with the signals’ assignment. In addition to the peaks relating to chloroform (CHCl_3_) and tetramethylsilane (TMS) at δ = 7.26 ppm and δ = 0 ppm, respectively, the following signals can be noted as follows:

δ = 7.18 ppm, which is given by the combination of two singlet signals relating to the protons 1 and 5 of the furan ring.

δ = 4.33 ppm, given by the overlap of two triplets relating to the 2 and 6 protons (in α position with respect to the carbonyl group) of hexanediol and pentanediol, respectively.

δ = 1.81 ppm, resulting from the overlapping of two multiplets relating to the protons 3 and 7 (in β position with respect to the carbonyl group) of hexanediol and pentanediol, respectively.

δ = 1.55 ppm, a multiplet relating to protons 8 of pentanediol.

δ = 1.47 ppm, a multiplet relating to protons 4 of hexanediol.

On the basis of the peak assignment described above, as any other significant peak is absent, it can be concluded that no secondary reactions occurred during the synthesis.

Subsequently, ^13^C-NMR analyses were carried out to better observe differences in the copolymers’ molecular architecture. As the number of transesterification reactions increases with reaction time (i.e., the length of the blocks decreases), the number of mixed forms increases at the expense of pure forms. Specifically, a pure form is defined as a chain segment in which the furan ring is bound only to hexanediol (H-F-H segments) or only to pentanediol (Pe-F-Pe segments) at both ends. On the other hand, a mixed form refers to a portion of the copolymer chain in which the furan rings are bound at one end to pentanediol and at the other end to hexanediol (H-F-Pe and Pe-F-H segments, respectively).

By analysing the intensity of these peaks, it is possible to calculate the length of the blocks of the different copolymers. In more detail, P_H-F-Pe_, the probability of finding a pentanediol subunit next to a hexanediol one, and P_Pe-F-H_, the probability of finding a hexanediol subunit next to a pentanediol one, can be calculated as follows:

$$P_{H\text{-}F\text{-}Pe}=\frac{(I_{H\text{-}F\text{-}Pe}+I_{Pe\text{-}F\text{-}H})/2}{{(I}_{H\text{-}F\text{-}Pe}+I_{Pe\text{-}F\text{-}H})/2+I_{H\text{-}F\text{-}H}}$$

$$P_{Pe\text{-}F\text{-}H}=\frac{(I_{H\text{-}F\text{-}Pe}+I_{Pe\text{-}F\text{-}H})/2}{(I_{H\text{-}F\text{-}Pe}+I_{Pe\text{-}F\text{-}H})/2+I_{Pe\text{-}F\text{-}Pe}}$$

where

I_H-F-Pe_ and I_Pe-F-H_ are the intensity of the signals related to the mixed forms, I_H-F-H_ is the intensity of the signal relating to the H-F-H pure form, while I_Pe-F-Pe_ is the intensity of the signal relating to the Pe-F-Pe pure form.

The block length was obtained as follows:

$$L_{F\text{-}H}=\frac{1}{P_{H\text{-}F\text{-}Pe}}$$

$$L_{F\text{-}Pe}=\frac{1}{P_{Pe\text{-}F\text{-}H}}$$

As to the degree of randomness (R), it ranges between 0 (for physical blends) and 1 (for random copolymers), while when it falls between 0 and 1, a block molecular architecture is expected. In more detail, the degree of randomness can be expressed as follows:

$$R=P_{H\text{-}F\text{-}Pe}+P_{Pe\text{-}F\text{-}H}$$

**Figure 2:**
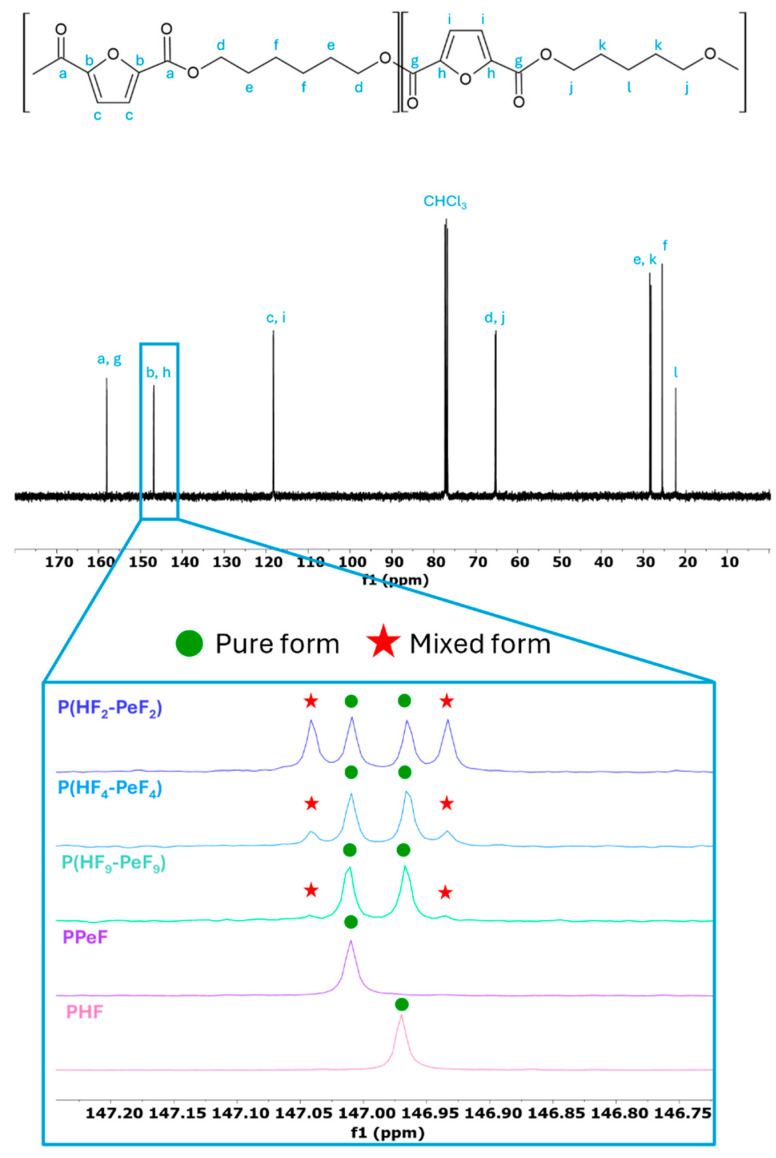
^13^C-NMR spectrum of P(HF_9_–PeF_9_) copolymer with peaks’ assignment and zooming of the zone of about 147 ppm to highlight the occurrence of mixed forms in the homopolymers and all copolymers under study.

The ^13^C-NMR spectrum of copolymer P(HF_9_–PeF_9_), with peak assignment, is shown in Figure 2. As observed, in addition to the signals relating to chloroform and tetramethylsilane at δ = 77 ppm and δ = 0 ppm, respectively, the following signals can be seen:

δ = 158.22 ppm and δ = 158.17 ppm, which correspond to the a and g carbonyl carbons.

δ = 146.97 and δ = 147.02, which correspond to b and h, relative to the carbons of the furan ring in α position with respect to the carbonyl group.

δ = 118.48 and δ = 118.41, which are the signals of the c and i carbons of the furan ring.

δ = 65.53 and δ = 65.33: signals d and l, relative to the carbons of hexanediol and pentanediol in α position with respect to the ester group, respectively.

δ = 28.60 and δ = 28.32: signals e and k, relative to carbons of hexanediol and pentanediol in β position with respect to the ester group, respectively.

δ = 25.64 ppm: signal of the f carbons of hexanediol.

δ = 22.44: signal of the l carbon of pentanediol.

Figure 2 also shows an enlargement of the area around δ = 147 ppm, where peaks b and h fall. As the reaction time increases, it is evident that the pure forms decrease in intensity compared to the mixed forms, which become progressively more intense. In parallel, block length decreases and R value increases (Table 1). As expected, for the copolymers P(HF_9_–PeF_9_) and P(HF_4_–PeF_4_) a block molecular architecture was confirmed (R values of 0.22 and 0.5, respectively), while in the case of P(HF_2_–PeF_2_) a random molecular architecture was obtained (R equal to 1).

All the polymers examined in this study were analysed by gel permeation chromatography to obtain the molecular weight (M_n_) and polydispersity index (Đ). As can be seen in Table 1, the molecular weights are high in all cases (above 40,000 g/mol) and quite comparable with each other. This further confirms good control over the synthetic process. As is well known, achieving a high molecular weight is essential for ensuring processable and good mechanical properties. The polydispersity indices obtained are in the range 1.4–1.7, typical of polyesters synthesised by melt polycondensation.

### 3.2. Thermal Characterisation

**Figure 3:**
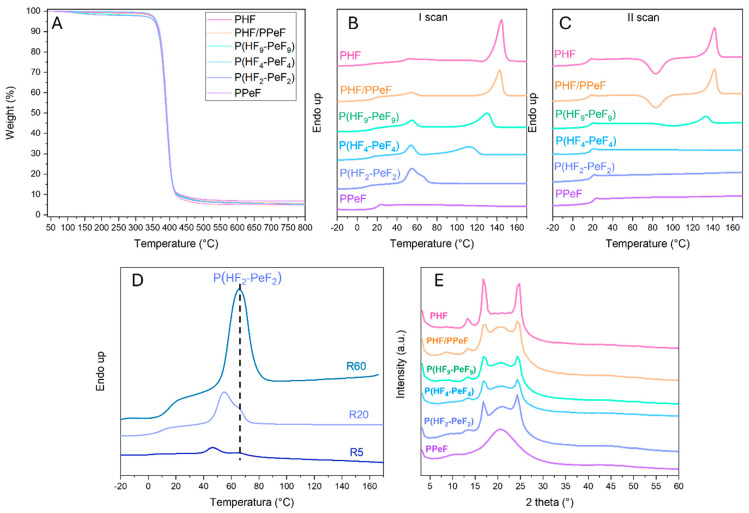
TGA curves (**A**), first (**B**), and second (**C**) DSC scans, comparison of three P(HF_2_–PeF_2_) DSC scans taken at different speeds (**D**), and WAXS profile (**E**) of PHF, PPeF, PHF/PPeF, and the copolymers.

The compression moulded film polymers under study were analysed by means of TGA to study their thermal stability and degradation temperatures. Figure 3A and Table 2 show the thermogravimetric curves and the relative thermal data, respectively. As can be seen, all the materials demonstrate high and comparable thermal stability, which is one of the strong points of furan-based polyesters [47,48,49,50,51,52]. In all cases, the T_onset_ value is above 370 °C, and the T_max_ value is in the 390–393 °C range. The degradation of all the materials occurs in a single step and, once 800 °C is reached, a residual char of approximately 5% of the initial weight is present.

The polymers under study were also thermally analysed by means of DSC. Figure 3B and Figure 3C show the first and second DSC scans, respectively, of all the materials, while Table 2 reports the relevant thermal data. As to the homopolymers, at room temperature PPeF is rubbery and completely amorphous, as evidenced by the presence of only a single endothermic jump at 18 °C due to glass-to-rubber transition. Conversely, PHF shows an intense melting peak at 144 °C, while the endothermic jump relative to the glass-to-rubber transition is barely visible, demonstrating that this material is characterised by high crystallinity. This behaviour is supported by a high melting enthalpy (ΔH_m_ of 38 J/g). A smaller endothermic peak can also be observed at around 50 °C, consistent with previous observations carried out on other FDCA-based polyesters [35]. The PPeF/PHF blend shows all the thermal transitions of the two reference homopolymers, including the baseline shift relative to the T_g_ of PPeF, the small endothermic peak around 50 °C, and an intense melting peak of PHF crystals, whose intensity is in line with the composition of the blend. For the three copolymers, there is only one T_g_ jump and one high melting peak, which decreases in intensity and temperature as the block length decreases (i.e., as the mixing time increases). This phenomenon is due to the formation of crystals with progressively lower degrees of perfection (lower T_m_) and a progressive decrease in the materials’ ability to crystallise (lower ΔH_m_). In the case of the random copolymer P(HF_2_–PeF_2_), the melting peak almost overlaps with the lower endothermic peak. On the other hand, the peak at around 50 °C increased considerably in intensity as the mixing time increased.

The effect of the heating rate on the two endothermic peaks was evaluated by conducing three thermal scans at different speeds (R5 = 5 °C/min, R20 = 20 °C/min, and R60 = 60 °C/min) on the P(HF_2_–PeF_2_) copolymer (Figure 3D). The position of the higher melting peak remains the same regardless of the scanning rate, whereas for the lower temperature, the peak changes. In more detail, compared to the curve measured at R20, in the scan carried out at the lowest speed (R5), the low temperature peak moved to lower temperatures, while at the highest speed (R60) it shifts to 63 °C, completely overlapping with the high melting endothermic peak. As is well known, the melting phenomenon is a first-order transition and therefore independent of the heating rate [53]. Conversely, a dependence on the scanning rate is indicative of a second-order transition, such as the isotropization of ordered phases (known as mesophase) with a degree of order lower than the common crystalline one [54,55,56]. This is in line with several studies in the literature on furan-based polymers [35,54,57,58]. The presence of this phase can also explain why PPeF, despite being amorphous and rubbery at room temperature, can be processed into self-sustaining films.

As to the second scan curves (Figure 3C), PPeF shows the same profile as the one already observed in the I scan. Conversely, PHF exhibits a T_g_ of 17 °C, indicating a rubbery amorphous phase, a crystallisation peak at T = 60 °C, and a melting one at 144 °C. Since ΔH_cc_ < ΔH_m_ (Table 2), the polymer was not quenched by the rapid cooling, indicating the ability to crystallise, which cannot be completely inhibited. The physical blend exhibits a behaviour similar to the one of PHF. However, in this case ΔH_cc_ = ΔH_m_, indicating that quenching was effective and that the presence of PPeF prevents the rapid crystallisation of PHF. Regarding the copolymers, they are all amorphous in the second scan, although they show a different capability to crystallise, which becomes progressively lower as the block length decreases. In fact, the copolymer with the longest blocks exhibits a crystallisation peak, followed by a melting one with the same intensity. In contrast, the other two copolymers are completely amorphous, displaying only the T_g_ jump, which is single, since the two parent homopolymers are characterised by very similar T_g_ values. As expected, the T_g_ values of the blend and all copolymers are similar.

### 3.3. Structural and Morphological Characterisation

Wide-angle X-ray diffractometric analysis (WAXS) was carried out to clarify the nature of the ordered phases of the materials under study. The diffractograms of the different samples are shown in Figure 3E, while the values of crystallinity degree are listed in Table 2. PHF is the polymer with the highest degree of crystallinity (X_c_ = 37%), with a diffractogram characterised by three intense diffraction peaks overlaid on the bell-shape profile related to the amorphous portion, in line with previously published papers [35,37,59]. In contrast, PPeF is completely amorphous, as its diffractogram only consists of the amorphous halo. All the other materials are semicrystalline and show the typical reflections of PHF. As expected, the physical blend shows a value of X_c_ which is about halved compared to that calculated for PHF homopolymer, in line with the composition. Conversely, the copolymers exhibited a decrease in crystallinity by decreasing the block lengths, in alignment with the calorimetric data (Table 2).

**Figure 4:**
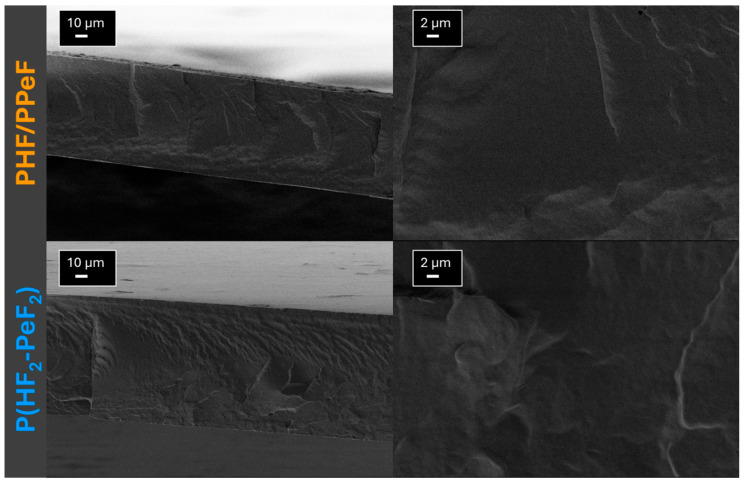
SEM micrographs of cryo-fractured cross-sections of PHF/PPeF (**top**) and P(HF_9_–PeF_9_) (**bottom**) at different magnifications (1000× on the **left**; 5000× on the **right**).

Scanning electron microscopy (SEM) was used to gain a better insight on the morphology of both the PHF/PPeF physical blend and the P(HF_2_–PeF_2_) copolymer. According to the pictures shown in Figure 4, the cross-sections of both materials are characterised by smooth and continuous surfaces, free from any significant voids, gaps, or phase separation. This uniform morphology clearly indicates the effective compatibility of the two reference homopolymers inside the blend and the successful chemical bonding in the copolymer.

### 3.4. Water Contact Angle Measurements

Surface wettability measurements were conducted on all the samples under study to determine the hydrophobicity of the film surface. Table 3 shows the contact angle values (WCAs), while in Figure S1 images of water droplets deposited on film surfaces are shown. As the measured angle is above the threshold of 90° in all cases, it can be concluded that all analysed polymers exhibit a hydrophobic character, with WCA values very similar to each other, ranging between 97° for PPeF and 100° for PHF.

### 3.5. Mechanical Characterisation

**Figure 5:**
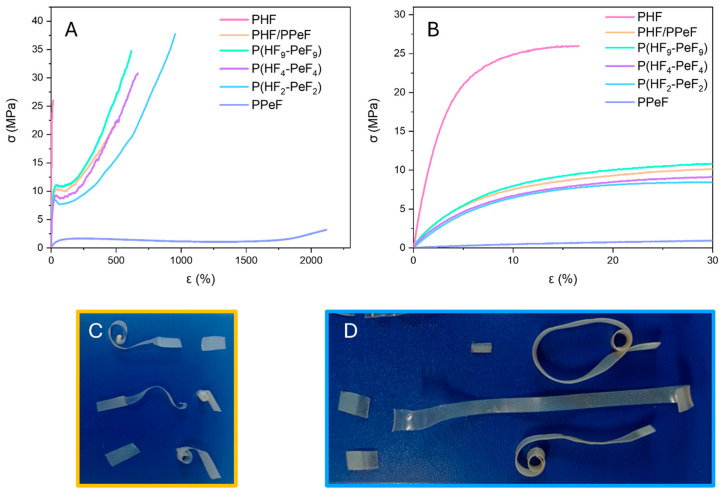
(**A**) Stress–strain curves of the materials under investigation; (**B**) zoom of the curves in the strain range of 0–30%. Rolling of the strips of (**C**) PHF/PPeF and (**D**) P(HF_2_–PeF_2_) after breaking.

Figure 5 shows the stress–strain curves of the materials which are the subject of this work, while Table 3 lists the relevant mechanical characterisation data. PHF is the most rigid material (E = 647 MPa) due to its high crystallinity, with a high stress at break (25 MPa) and the lowest strain at break (17%). On the other hand, PPeF is characterised by the lowest elastic modulus (60 MPa), together with the lowest stress at break (2.5 MPa), and an exceptional strain at break, higher than 2000%. Additionally, no yield phenomenon was observed in the stress–strain curve of PPeF, as expected for an elastomer. As previously reported, the mechanical response can be explained as due to the presence of a mesophase originated from inter-chain hydrogen bonds as well as π–π interactions [36]. The other materials exhibit intermediate mechanical behaviour compared to the two homopolymers. In more detail, the longer block copolymers show very similar elastic moduli (166–184 MPa), which become slightly lower and closer to that of PPeF for the random copolymer (111 MPa) (Figure 5B). An opposite trend was observed for the stress and the strain at break, which ranged from 31 MPa and 479% for P(HF_9_–PeF_9_) to 42 MPa and 950% for P(HF_2_–PeF_2_), respectively. Excluding the effect of molecular weight (as the M_n_ values are high and comparable in all cases, see Table 1) and amorphous phase mobility (similar and well below room temperature in all cases, see Table 2), the observed differences can be explained by the different block lengths and, in turn, by the different crystallinity degree (Table 2). Interestingly, the physical blend exhibits a mechanical response which is intermediate with respect to PPeF and PHF. In particular, the elongation at break reached almost 500%, a further proof of the good compatibility between the reference homopolymers, in agreement with the SEM analysis (Figure 4). Last, all the materials show yield at strain values below 50%.

Of note, tensile tests revealed that film strips underwent rolling and rapidly returned to a shape similar to their original one upon breakage (Figure 5C,D, Video S1). This finding confirms the potential of these materials for fabricating elastic and highly stretchable sutures.

**Figure 6:**
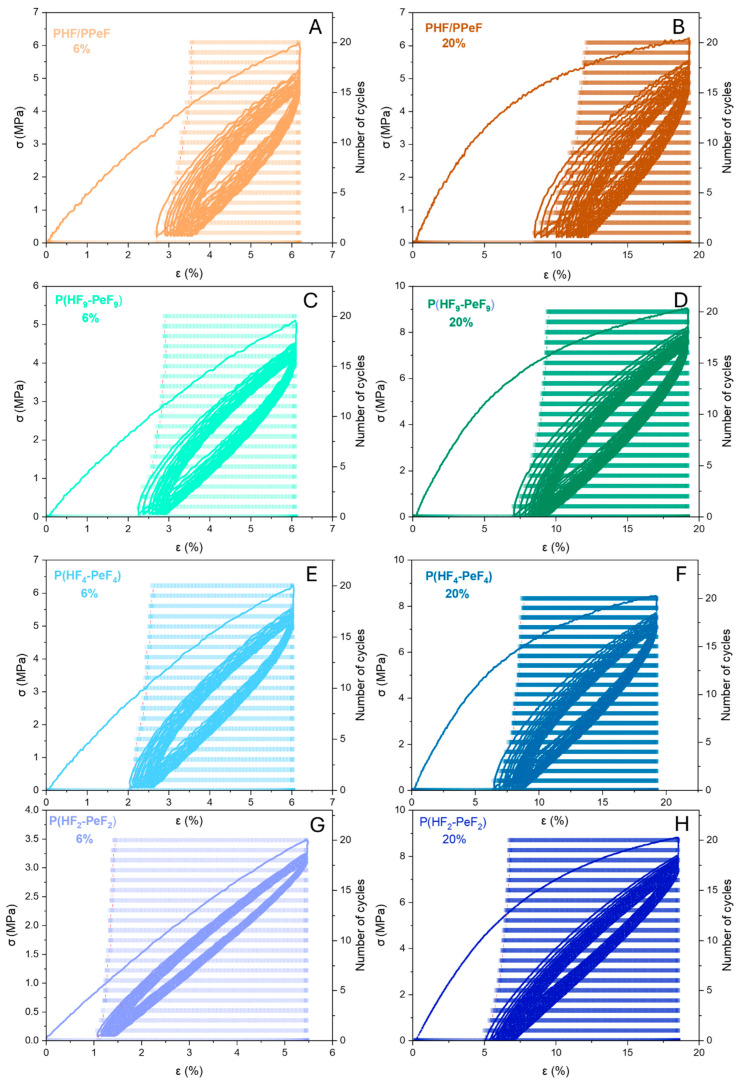
Stress–strain curves obtained from cyclic testing of PHF/PPeF at (**A**) 6% and (**B**) 20% of maximum elongation; P(HF_9_–PeF_9_) at (**C**) 6% and (**D**) 20% of maximum elongation; P(HF_4_–PeF_4_) at (**E**) 6% and (**F**) 20% of maximum elongation; P(HF_2_–PeF_2_) at (**G**) 6% and (**H**) 20% of maximum elongation.

To quantitatively evaluate the recovery properties of the materials under study, cyclic tests were also carried out (Figure 6), except for PHF, due to its poor elongation at break, and for PPeF, due to its very low stress values. Two separate tests were carried out for each material, at two different maximum strain values (6% and 20%), in order to test the specimens in both the elastic region and in a point further away from the initial linear stretch.

The load–unload cycles are all characterised by low hysteresis and high elasticity, even after 20 cycles, with the highest recoveries when the lowest elongation was applied. For each material, different values of elastic return were observed, with the highest in the first load cycle and the lowest in the final cycle. This confirms that the materials’ ability to recover their original dimensions decreases as the number of cycles increases. However, an asymptotic trend in the elastic return values was observed for all the polymers as the number of cycles increased, with a residual deformation plateau being reached. When the different materials are compared, it can be seen that the hysteresis areas decrease (i.e., % recovery increases) as the block length is decreased, ranging from 50% for the physical blend to over 70% for the P(HF_2_–PeF_2_) random copolymer.

### 3.6. Hydrolytic Degradation Tests

The PHF/PPeF blend and P(HF_2_–PeF_2_) copolymer were subjected to in vitro hydrolytic degradation experiments under physiological temperature (37 °C) and pH (7.4), in order to confirm their applicability as non-bioresorbable materials. The choice of these two materials was based on their ease scale up preparation at industrial level and their optimal mechanical properties for the intended device.

According to gravimetric weight loss measurements, made after six months of incubation, the weights of both the blanks and the incubated samples remained practically unchanged over time. Indeed, the PHF/PPeF blend showed a gravimetric weight loss of 1.28 ± 0.01% and the P(HF_2_–PeF_2_) copolymer showed a gravimetric weight loss of 0.40 ± 0.01%. Molecular weight measurements revealed that the copolymer lost about 15 ± 3% of its initial weight after 6 months.

In order to verify how the hydrolytic attack might have affected the crystallinity and the main thermal transitions of the materials under investigation, I DSC scan analyses were carried out (Table S1, Figure S2). As to the PHF/PPeF blend, the profiles of blank and partially degraded samples are similar and comparable to the one before degradation, only a shift to higher temperature of the lower endothermic peak (from 54 °C to 77 °C), being observed. However, its intensity remained almost equal.

In the P(HF_2_–PeF_2_) copolymer, the two partially overlapping endothermic peaks were shifted towards higher temperatures (55 °C to 75 °C) and became slightly more intense, both in the blank and in the partially degraded samples, indicating an improvement and a slight increase in the crystalline portion.

Strain–stress tests were performed on partially degraded samples and their corresponding blanks to determine if the polymer’s mechanical properties remained unchanged after six months of incubation. Table S1 gives the values of Young’s modulus and stress and strain at break, while the corresponding stress–strain curves are shown in Figure S2.

As can be seen, the PHF/PPeF physical mixture maintains an unchanged Young’s modulus, which indicates that the material did not lose its tensile strength. However, the stress at break decreased from 21 MPa to 11 MPa, as did the strain at break. This is probably related to an improvement in the crystalline phase perfection, as observed through DSC analysis.

Conversely, the elastic modulus of the P(HF_2_–PeF_2_) copolymer increased from 111 MPa of the sample at time zero to 170 MPa of the blank and to 190 MPa of the partially incubated sample; in parallel, stress at break was almost halved, while strain at break remained almost unaltered, as a result of the refinement of the crystalline portion.

### 3.7. Citotoxicity Tests

#### 3.7.1. Direct Contact Test

**Figure 7:**
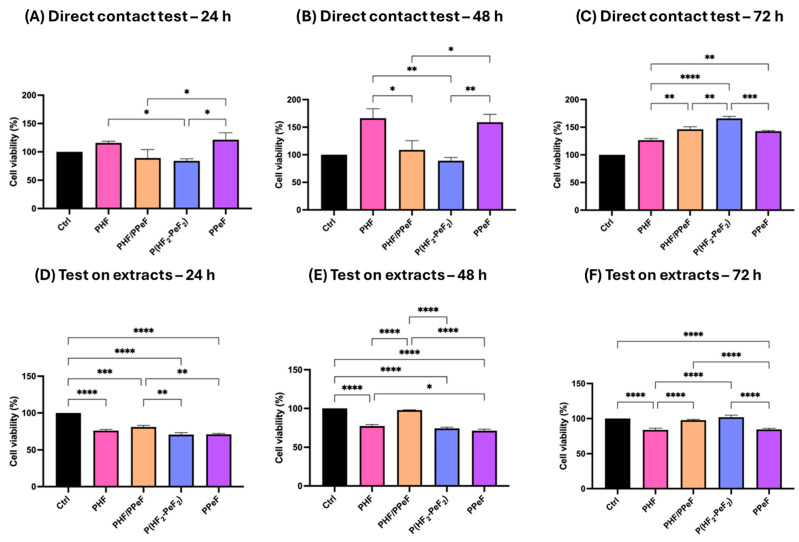
Cell viability of HCornF cells in contact with PHF, PPeF, PHF/PPeF blend, and P(HF_2_–PeF_2_) copolymer for 24 h (**A**), 48 h (**B**), 72 h (**C**). Extract test of PHF, PPeF, PHF/PPeF blend, and P(HF_2_–PeF_2_) copolymer carried out for 24 h (**D**), 48 h (**E**), and 72 h (**F**) on HCornF. All the values were expressed as percentage ± SD (data obtained from 3 experiments) and normalised to untreated controls (cells with culture medium) analysed by One-way Anova test with Tukey’s multiple comparison. Results of *p* < 0.05 were considered significant (* *p* < 0.05; ** *p* < 0.01; *** *p* < 0.001; **** *p* < 0.0001).

To evaluate the direct effect and possible toxicity of the PHF and PPeF homopolymers, the PHF/PPeF blend, and the P(HF_2_–PeF_2_) copolymer on HCornF cells, direct contact tests were performed. As to the homopolymers, at 24 h they exhibited viability levels comparable to those of the control group (Figure 7A), increasing for the following timepoints (Figure 7B,C), indicating a time-dependent enhancement in cell metabolic activity. Although the initial viability at 24 and 48 h of PHF/PPeF and P(HF_2_–PeF_2_) appeared to induce a slowing effect on mitochondrial activity, at 72 h there was a significant growth of cells in contact with both the materials, especially for the copolymer, suggesting a very good time-dependent adaptation (Figure 7C). Last, in all the cases, and for all the timepoints considered, all the experimental groups showed more than 70% cell viability, which is the threshold value to assess that materials are not cytotoxic.

#### 3.7.2. Extract Tests

The capability to sustain the cell viability of PHF, PPeF, PHF/PPeF, and P(HF_2_–PeF_2_) was evaluated also through in vitro tests on extracts. As to cell viability (Figure 7D–F), all the experimental groups showed more than 70% cell viability at all the different timepoints, confirming their no-cytotoxicity. In more detail, the results obtained at 24 h demonstrated a slight decrease in cell viability in comparison to the control samples, with an inversion of this trend for the following timepoints. As to the homopolymers, compared to the previous tests, a lower viability was observed, although in all the cases well above the threshold level. Moreover, at 48 h cells incubated with the PHF/PPeF extracts reached a viability higher than 90%. The same viability was reached also for P(HF_2_–PeF_2_) after 72 h of incubation, indicating a very good ability of these materials to sustain cell proliferation.

## 4. Conclusions

The need for improved ophthalmic devices is urgent, and this demand is predicted to continuously grow in the coming years. Despite advances in surgical techniques, issues due to unstable tissue positioning often lead to postoperative complications. Shape-memory materials such as NiTinol^TM^, which is widely used in other fields, have inspired new solutions in this regard. However, the mechanical properties of these new materials should be properly tailored, as they must suit the proper resistance of the delicate ocular tissues. Considering this complex scenario, two aromatic homopolymers, PPeF, a highly deformable but low-strength elastomer, and PHF, highly rigid and semicrystalline, were successfully physically and chemically mixed to obtain materials suitable for biomedical purposes for the first time. Copolymers with the same weight composition but different molecular architecture were prepared through reactive blending, and the different molecular architecture allowed to finely tune the thermal, structural, and mechanical properties. First, all the materials demonstrated very high thermal stability, comparable to one of the homopolymers, which ensures a wide processing window and suggests that the physical and chemical modifications did not alter one of the strong points of the starting materials. Moreover, the physical blend and the copolymers exhibited superior mechanical strength compared to PPeF, lower stiffness than PHF, and high deformation at break. Of note, after breaking they exhibited the high recovery typical of PPeF, which was confirmed also after cyclic tests, proving their great elasticity and stretchability. In terms of hydrolytic stability, the results were very promising, as both the physical blend and the random copolymer did not show any appreciable weight loss and maintained their thermal and mechanical features. In terms of biological properties, which were tested with human corneal fibroblasts, both the materials investigated exhibited no-cytotoxic behaviour, with cells being able to survive during the analysis, indicating that the environment created was suitable for cell maintenance. All these results confirm the real applicability of these materials for applications as superelastic sutures in the field of ophthalmic surgery.

## Figures and Tables

**Table 1:** Molecular characterisation data (^13^C-NMR and GPC) of PHF, PPeF, and their copolymers.

	^13^C-NMR			GPC	
	L_H-F_	L_Pe-F_	R	M_n_ (g/mol)	Đ
PHF	-	-	-	43,900	1.7
P(HF_9_–PeF_9_)	9	9	0.22	46,000	1.7
P(HF_4_–PeF_4_)	4	4	0.5	49,700	1.5
P(HF_2_–PeF_2_)	2	2	1	69,400	1.6
PPeF	-	-	-	52,600	1.4

**Table 2:** Thermal (TGA and DSC) and structural (WAXS) characterisation data of PHF, PPeF, PHF/PPeF, and the copolymers.

	DSC										TGA		WAXS
	I Scan				II Scan								
	T_g_ (°C)	ΔC_p_ (J/g*°C)	T_m_ (°C)	ΔH_m_ (J/g)	T_g_ (°C)	ΔC_p_ (J/g*°C)	T_cc_ (°C)	ΔH_cc_ (J/g)	T_m_ (°C)	ΔH_m_ (J/g)	T_onset_ (°C)	T_max_ (°C)	X_c_ (°C)
PHF	n.d.	n.d.	53 144	1.4 38	17	0.258	60	23	144	35	375	393	37 ± 4
PHF/PPeF	17	0.433	54 143	3.6 26	17	0.567	83	25	144	25	370	390	20 ± 4
P(HF_9_–PeF_9_)	17	0.203	54 131	6.3 20	17	0.382	101	7	133	7	369	390	19 ± 4
P(HF_4_–PeF_4_)	18	0.305	54 108	9 13	18	0.347	-	-	-	-	373	390	16 ± 3
P(HF_2_–PeF_2_)	18	0.433	55 63	17 4	18	0.481	-	-	-	-	373	391	11 ± 3
PPeF	18	0.464	-	-	18	0.579	-	-	-	-	370	391	0

**Table 3:** Mechanical characterisation data and WCA values of PHF, PPeF, PHF/PPeF, and copolymers.

	E (MPa)	σ_b_ (MPa)	ε_b_ (%)	WCA (°)
PHF	674 ± 46	25 ± 1	17 ± 2	100 ± 2
PHF/PPeF	184 ± 34	21 ± 5	479 ± 65	98 ± 2
P(HF_9_–PeF_9_)	166 ± 14	31 ± 2	612 ± 57	97 ± 2
P(HF_4_–PeF_4_)	182 ± 41	32 ± 5	666 ± 71	97 ± 3
P(HF_2_–PeF_2_)	111 ± 12	42 ± 5	950 ± 93	99 ± 2
PPeF	60 ± 13	2.5 ± 0.7	2025 ± 183	97 ± 2

## Data Availability

Raw data are available from the corresponding authors upon reasonable request.

## Supplementary Materials

The following supporting information can be downloaded at: https://www.mdpi.com/article/10.3390/polym17233220/s1, Figure S1: Images of water droplets immediately after deposition on film surfaces of PHF, PPeF the blend and the copolymers; Figure S2: First DSC scan of partially degraded samples (degr), compared of the neat (t0) and blank samples of (A) PHF/PPeF; (B) P(HF2-PeF2); Stress-strain curves of PHF/PPeF partially degraded samples (degr), compared to the neat (t0) and blank samples of (C)PHF/PPeF; (D) P(HF2-PeF2); Table S1: Thermal (DSC) and mechanical characterisation data of the partially hydrolysed PHF/PPeF and P(HF2-PeF2) samples (degr) compared with those of the non-hydrolysed (t0) samples and the corresponding blanks; Video S1: PHF/PPeF blend mechanical test, with a focus on the immediate return and simultaneous rolling after the sample breaks.
