# Supplementary material for: Novel Superelastic Polyesters Based on 2,5-Furandicarboxylic Acid for Potential Use in Ophthalmic Surgery
Source: Polymers (Basel). 2025 Dec 3;17(23):3220. doi: 10.3390/polym17233220 (PMC12694372; doi:10.3390/polym17233220)
Supplement: Supplementary file 1 [file polymers-17-03220-s001.zip › polymers-3976952-supplementary.pdf]

SUPPLEMENTARY MATERIALS

WATER CONTACT ANGLE MEASUREMENTS

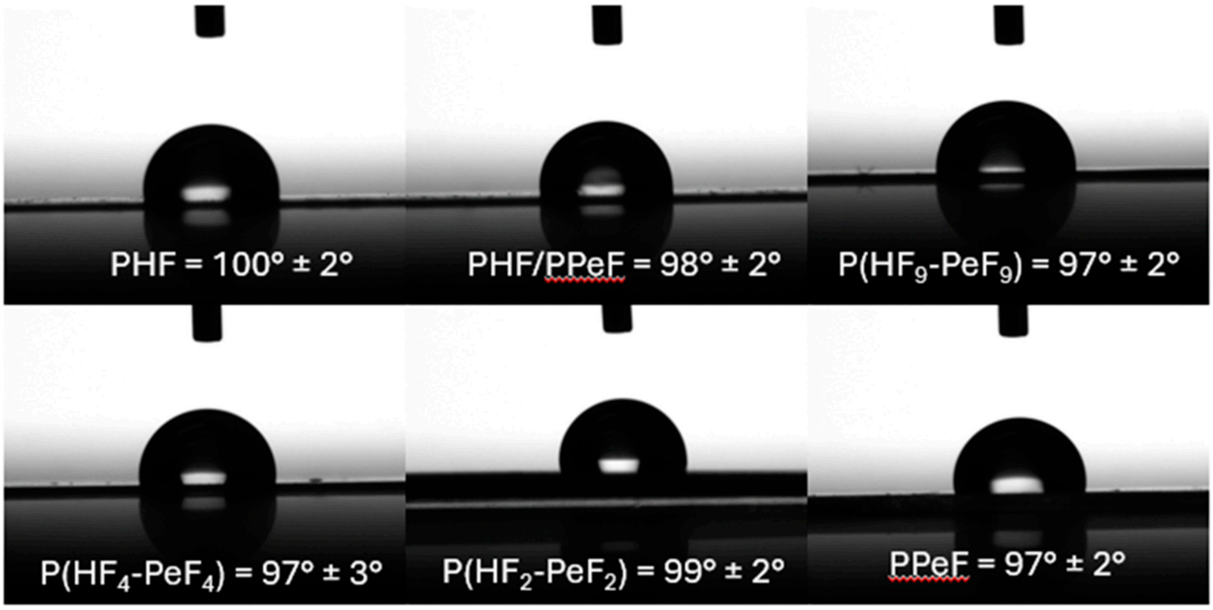

Figure S1. Images of water droplets immediately after deposition on film surfaces of PHF, PPeF the blend and the copolymers.

MECHANICAL CHARACTERISATION

PHF-PPeF mechanical test.mp4

Video S1. PHF/PPeF blend mechanical test, with a focus on the immediate return and simultaneous rolling after the sample breaks.

HYDROLYTIC DEGRADATION TESTS

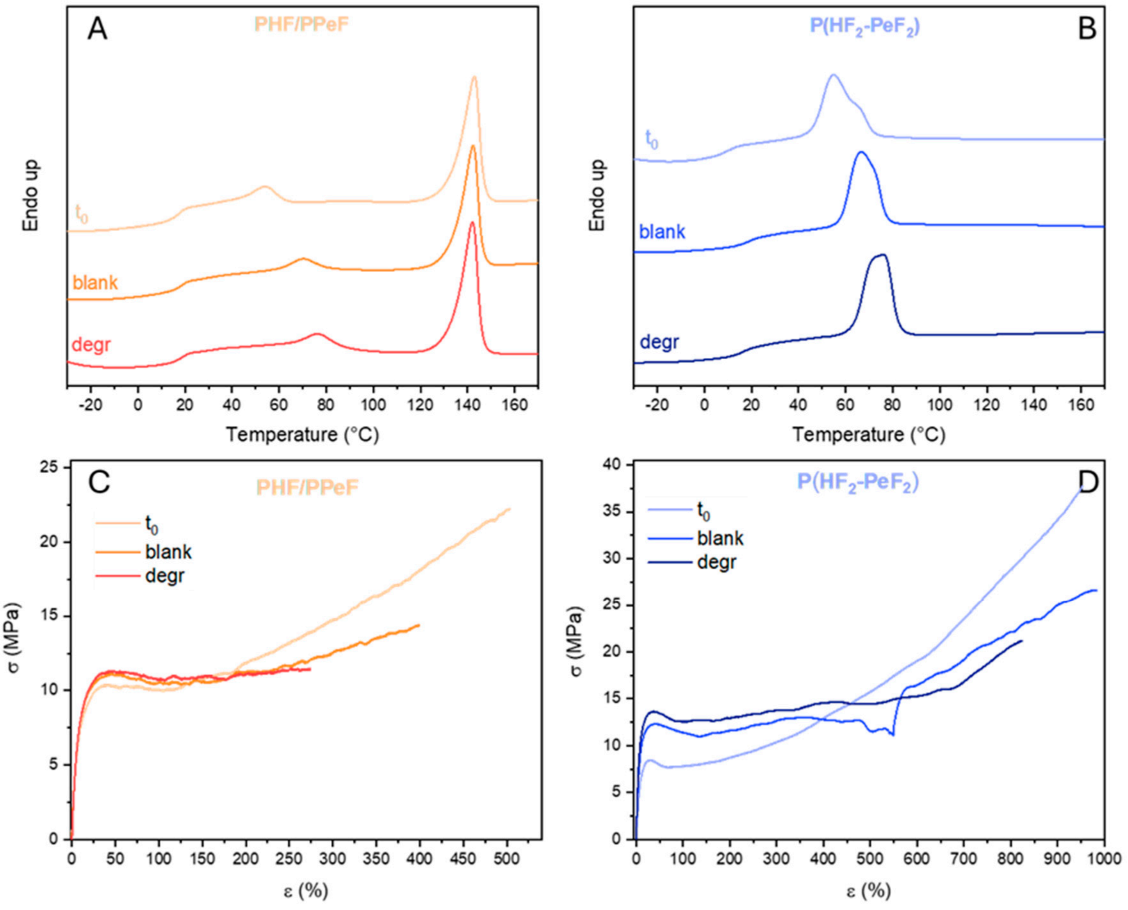

Figure S2. First DSC scan of partially degraded samples (degr), compared of the neat (t_0) and blank samples of (A) PHF/PPeF; (B) P(HF_2-PeF_2); Stress-strain curves of PHF/PPeF partially degraded samples (degr), compared to the neat (t_0) and blank samples of (C)PHF/PPeF; (D) P(HF_2-PeF_2).

Table S1. Thermal (DSC) and mechanical characterisation data of the partially hydrolysed PHF/PPeF and P(HF_2-PeF_2) samples (degr) compared with those of the non-hydrolysed (t_0) samples and the corresponding blanks.

	T_g (°C)	ΔC_p (J/g*°C)	T_{m1} (°C)	ΔH_{m1} (J/g)	T_{m2} (°C)	ΔH_{m2} (J/g)	E (MPa)	σ_b (MPa)	ε_b (%)
PHF/PPeF t_0	17	0.433	54	3.6	143	26	184±34	21±5	479±65
PHF/PPeF blank	18	0.268	70	3.3	142	26	186±10	12±2	399 ± 87
PHF/PPeF degr	18	0.264	77	3.3	142	28	181±20	11±1	170±41
P(HF_2-PeF_2) t_0	16	0.481	55	17	63	4	111±12	42±5	950±93
P(HF_2-PeF_2) blank	18	0.253	-	-	67	24	174±21	26±1	867±95
P(HF_2-PeF_2) degr	18	0.293	-	-	75	27	190±26	26±4	903±98
